# Severe odontogenic infections with septic progress – a constant and increasing challenge: a retrospective analysis

**DOI:** 10.1186/s12903-019-0866-6

**Published:** 2019-08-02

**Authors:** H. Weise, A. Naros, C. Weise, S. Reinert, S. Hoefert

**Affiliations:** 1 Department of Oral and Maxillofacial Surgery, University Hospital Tuebingen, Osianderstrasse 2-8, 72076 Tübingen, Germany; 2 Department of Orthodontics, University Hospital Tuebingen, Osianderstrasse 2-8, 72076 Tübingen, Germany

**Keywords:** Odontogenic infection, Sepsis, Antibiotic therapy, septicaemia, hospital care

## Abstract

**Background:**

More than 90% of all infections in the head and neck region can be traced back to an odontogenic origin. In rare cases they can lead to sepsis, which may pose a vital threat to the patient. The purpose of this study was to analyse characteristics concerning etiology and progress of severe odontogenic infections with a fulminant development.

**Methods:**

All patients with odontogenic infections requiring hospital admission were included in a retrospective analysis conducted from 02/2012 to 09/2017. Of 483 patients 16 patients (13 male, 3 female) showed severe exacerbation with septic progress. The average age was 52.8 years. All patients underwent at least one surgical procedure that involved an extraoral incision and drainage as well as high volume irrigation intraoperatively. At least one revision was required for four of the patients. Three patients showed an exceedingly severe disease progression with multiorgan dysfunction syndrome (MODS) and circulatory arrest. Antibiotic treatment was adjusted according to the results of an antibiogram and resistogram. Irrigation with saline was done several times a day.

**Results:**

Sixteen patients showed odontogenic infections that spread over multiple maxillo-facial and cervical regions accompanied by septic laboratory signs. All these patients needed intensive care and a tracheostomy. The hospitalization period was 27.8 days on average. In 16 cases risk factors for the development of odontogenic abscesses like diabetes mellitus, obesity, chronic alcohol and nicotine abuse, rheumatism and poor oral hygiene were present. Intraoperative swabs showed a typical polymicrobial aerobic and anaerobic spectrum of oral bacteria, especially anaerobes and streptococci, mainly *Streptocococcus viridans*.

**Conclusion:**

Odontogenic infections with fulminant progression should be treated based on clinical and imaging data with immediate surgical incision and drainage including elimination of odontogenic foci as well as intensified intra- and postoperative irrigation. If needed, repeat imaging followed by further incisions should be performed. Immediate antibiotic treatment adapted to the antibiogram is of utmost importance. A combination of tazobactam and piperacillin has proven to be a good first choice and can be recommended for abscesses that spread over multiple levels with initial signs of severe infections.

## Background

The majority of head and neck infections are odontogenic [17]. Odontogenic infections can spread and cause severe complications, e.g. compromised airways, sepsis, tissue necrosis, endocarditis, mediastinitis and deep neck infections [3]. These severe odontogenic infections can be potentially life-threatening [14, 17]. There are several predisposing factors which may exacerbate odontogenic infections, such as immunodeficiency (human immunodeficiency virus HIV), long-term diabetes mellitus, obesity, chronic alcohol abuse, hepatitis, liver cirrhosis, immunosuppression after organ transplantation, chemotherapy, radiotherapy and systemic lupus erythematosus [4, 9, 11, 12, 16]. Usually odontogenic infections respond well to a combination of surgical sanitation, incision, drainage and antibiotic therapy [5, 14]. Odontogenic infections exhibit a variety of different pathogens: Streptococci, especially *Streptococcus viridans*, a representative of gram-positive aerobic bacteria and *Prevotella species*, a gram-negative anaerobic bacteria are common pathogens in odontogenic infections [1, 8, 15]. The purpose of this study was to analyse etiology and sequence of especially severe manifestations of odontogenic abscesses.

## Methods

This retrospective study included all 483 patients with odontogenic infections, who were hospitalized from February 2012 to September 2017 at the Department of Oral and Maxillofacial surgery, University Hospital Tuebingen, Germany. This time period was chosen because we found an increase of septic occurrences of odontogenic infections between these dates. The treatment for all patients included incision, drainage and surgical sanitation of the odontogenic focus plus antibiotic treatment. Intraoperative swabs were taken to adjust antibiotic treatment following performance of an antibiogram and a resistogram. Within this population, 16 patients (13 male, three female) with an average age of 52.75 (SD 9.5) exhibited critical courses and required postoperative intensive medical care. Nine patients underwent preoperative computed tomography (CT) to obtain an exact overview of the extent of infection. We confirm that we have read the Helsinki Declaration and have followed the guidelines in this investigation. This study has been approved by the local ethical committee.

## Results

All 16 patients suffered from pre-existing conditions, some of which may have contributed as predisposing factors to odontogenic abscesses and a systemic inflammatory reaction. These include, for example, diabetes mellitus, obesity, chronic alcohol and nicotine abuse, rheumatism, cardiological and neuro-psychiatric disorders and poor oral hygiene. A decayed mandibular molar or premolar was the infectious focus in all 16 patients (Table 1). All patients showed swelling of affected areas, trismus, dysphagia, dyspnea, involvement of several fascial spaces, phlegmonous spread, laboratory parameters of septicemia and a C-reactive protein (CRP) above 200 mg/l with white blood cell counts greater than 19*1000/ μl at admittance. Preoperatively white blood cell count averaged 22.0 ± 10.6*1000 cells/μl and CRP 251 ± 1.0 mg/ml. The preoperative CT scan of the head and neck region in nine patients provided an exact overview of the extent of infection and the affected fascial spaces. An antibiotic therapy with Clindamycin 600 mg 1–0-1 had been administered to three patients before hospital admission and surgical sanitation of the odontogenous focus had been performed. All Patients underwent extraoral incision, drainage of affected areas and elimination of odontogenic foci under general anaesthesia. On average, the maximum number of drainage tubes inserted was 9.1 ± standard deviation. A second look surgery was required in four patients. Ultimately, two patients required four surgical operations and another two cases required six operations.

**Table 1:** Overview of patients with severe odontogenic infections with septic progression

No.	Etiology (tooth)	Risk factors/ comorbidities	Therapy	Complications
1	35	obesity, nicotine abuse, poor oral hygiene	tooth removal, extraoral incision and drainage, revision (6x), tracheostomy	sepsis, acute respiratory insufficiency
2	47	obesity, diabetes mellitus, nicotine abuse, alcohol abuse, depression, poor oral hygiene	tooth removal, extraoral incision and drainage, tracheostomy	sepsis, MODS, acute respiratory insufficiency
3	36	nicotine abuse, depression, poor oral hygiene	tooth removal, extraoral incision and drainage, tracheostomy	sepsis, acute respiratory insufficiency
4	46	obesity, diabetes mellitus, COPD, nicotine abuse, poor oral hygiene	tooth removal, extra−/intraoral incision and drainage, revision (4x), tracheostomy	sepsis, MODS, circulatory arrest, acute respiratory insufficiency
5	46	diabetes mellitus, COPD, poor oral hygiene	tooth removal, extraoral incision and drainage, tracheostomy	sepsis, acute respiratory insufficiency
6	36	rheumatism, chronic heart failure	tooth removal, extra−/intraoral incision and drainage, revision (4x), tracheostomy	sepsis, MODS, circulatory arrest, acute respiratory insufficiency
7	35	Nicotine abuse, poor oral hygiene	tooth removal, extraoral incision and drainage, tracheostomy	sepsis, acute respiratory insufficiency
8	37	obesity, diabetes mellitus, alcohol abuse, nicotine abuse, poor oral hygiene	tooth removal, extraoral incision and drainage, tracheostomy	sepsis, acute respiratory insufficiency
9	36	depression, poor oral hygiene	tooth removal, extraoral incision and drainage, tracheostomy	sepsis, acute respiratory insufficiency
10	46	obesity, diabetes mellitus, poor oral hygiene	tooth removal, extra−/ intraoral incision and drainage, tracheostomy	sepsis, acute renal failure,acute respiratory insufficiency
11	34	obesity, poor oral hygiene	tooth removal, extraoral incision and drainage, tracheostomy	sepsis, acute respiratory insufficiency
12	47	depression, nicotine abuse, NSAR abuse, poor oral hygiene	tooth removal, extra−/ intraoral incision and drainage, revision (6x), tracheostomy	sepsis, MODS, circulatory arrest, acute respiratory insufficiency
13	46	nicotine abuse, poor oral hygiene	tooth removal, extraoral incision and drainage, tracheostomy	sepsis, acute respiratory insufficiency
14	46	depression, nicotine abuse, NSAR abuse, poor oral hygiene	tooth removal, extraoral incision and drainage, tracheostomy	sepsis, MODS, acute respiratory insufficiency
15	36	nicotine abuse, poor oral hygiene	tooth removal, extra−/ intraoral incision and drainage, tracheostomy	sepsis, acute respiratory insufficiency
16	35	COPD, nicotine abuse, poor oral hygiene	tooth removal, extraoral incision and drainage, tracheostomy	sepsis, acute respiratory insufficiency

Nine patients received an initial, carefully calculated, intravenous antibiotic therapy of cefuroxim 1.5 g 3x /d and likewise seven patients receiving ampicillin/sulbactam 3 g 3x/d immediately after hospital admission. Intraoperative swabs were taken by inserting the swab into the abscess space. These swabs showed a typical polymicrobial aerobic and anaerobic spectrum of oral bacteria. *Streptococcus viridans* was the predominant bacterium (75%), followed by *Staphylococcus epidermidis* (38%), *Enterococcus faecalis* (31%) and *Prevotella oris* (31%) (Table 2). All antibiograms and resistograms included the following antibiotics: ampicillin, ceferoxime, clindamycin, erythromycin, penicillin, piperacillin and tazobactam. Clindamycin had an increased mechanism of resistance to all groups of microrganisms whereas tazobactam and piperacillin showed susceptibility to all isolated bacteria (Table 3).

**Table 2:** Bacteria cultured from odontogenic infections with septic progress

Bacteria	No of patients (%)
Gram-positiv aerobic bacteria	
*Streptococcus viridans*	12 (75%)
*Staphylococcus epidermidis*	6 (38%)
*Streptococcus constellatus*	3 (19%)
*Streptococcus salvarius*	2 (13%)
*Streptococcus oralis*	1 (6%)
*Enterococcus faecalis*	5 (31%)
Gram-negative aerobic bacteria	
*Neisseria subflavia*	2 (13%)
*Escheria coli*	1 (6%)
Gram-negative anaerobic bacteria	
*Prevotella oris*	5 (31%)
*Haemophilus haemolyticus*	3 (19%)
*Porphyromonas gingivalis*	2 (13%)
Gram-positive anaerobic bacteria	
*Actinomyces meyeri*	3 (19%)
*Peptostreptococcus micros*	1 (6%)

**Table 3:** Susceptibility and resistence of isolated pathogens from odontogenic infections

Antibiotics	Number of isolates susceptible for	Number of isolates resistant to
Ampicillin	14 (87%)	2 (13%)
Ceferoxime	13 (81%)	3 (19%)
Clindamycin	10 (62%)	6 (38%)
Erythromycin	11 (69%)	5 (31%)
Penicillin	12 (75%)	4 (25%)
Piperacillin/Tazobactam	16 (100%)	0 (0%)

**Fig. 1:**
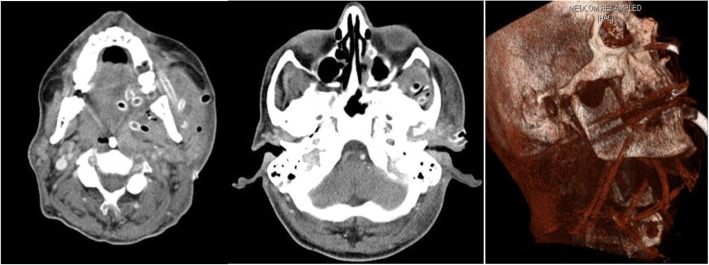
**a**-**b** Postoperative CT series in planar depiction after drainage with drains at lingual and buccal sides of the mandible. **c** CT 3D reconstruction after drainage with 9 tubes All areas with imaging correlated signs

**Fig. 2:**
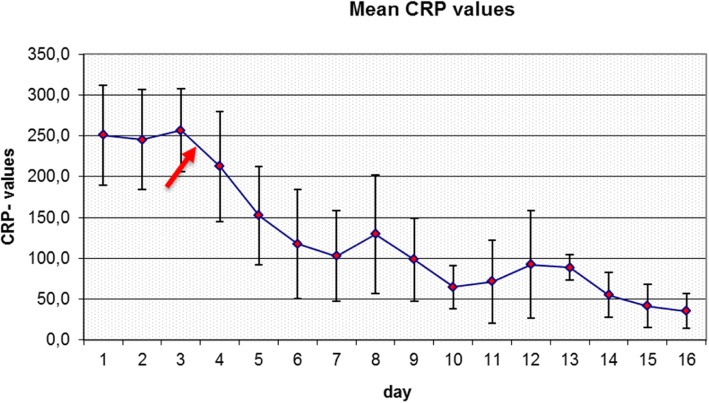
Course of the average CRP [mg/l] for all patients with standard deviation. The decrease after the initialization of the tazobactam/piperacillin therapy on the second day is noticeable (pointer)

**Fig. 3:**
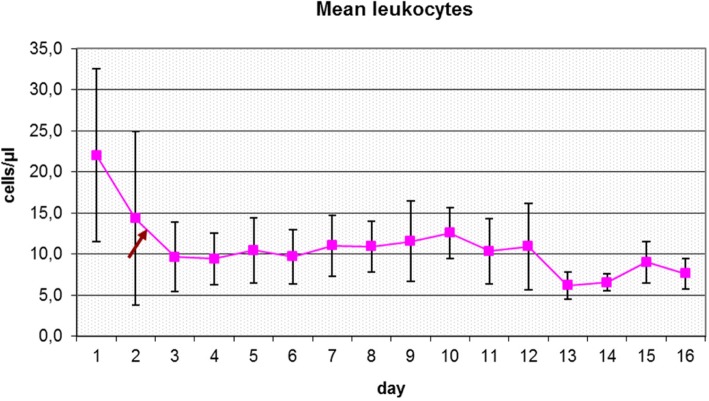
Course of the mean white blood cell count [cells/μl] of all patients with standard deviation.The decrease after the initialization of the tazobactam/piperacillin therapy on the second day is noticeable (pointer)

In all 16 patients, postoperative intensive medical care was required. The average length of postoperative medical care was 20.3 days (ranging from 8 to 33 days). All patients needed a temporary tracheostomy to secure airway management. The mean postoperative artificial respiration time was 15.6 days (ranging from 6 to 27 days). The average length of inpatient stay was 27.8 days (ranging from 11 to 40 days). Five patients had multi organ dysfunction syndrome (MODS) and circulatory arrest. The abscess cavity was irrigated multiple times a day (mean 2.3/die) with 100 ml or more saline 0.9% (Fig. 1). Based on the swab results, antibiotic treatment was adjusted to the antibiogram in all cases. After an average of 2.3 days, antibiotic treatment with tazobactam and piperacillin was started for all patients showing signs of a septic course. The mean time for the CRP parameter to decrease was during the third inpatient day (Fig. 2). Generally, the white blood cell count dropped on the first postoperative day (Fig. 3).

## Discussion

Odontogenic abscesses with fulminant progression that lead to severe, sometimes life-threatening complications like sepsis, airway obstruction, fasciitis, tissue necrosis, acute respiratory distress syndrome (ARDS), thrombosis, mediastinitis, and multiorgan dysfunction syndrome (MODS) are rare, but require extensive interdisciplinary collaboration especially with specialists for anesthesiology and intensive care medicine. Some predisposing factors or co-morbidities, e.g. diabetes mellitus, obesity, poor oral hygiene, and long-term nicotine or alcohol abuse may aggravate such septic progressions. These cases show greatly increased inpatient stays combined with a worse prognosis compared to patients who do not exhibit the named factors [7, 12, 13].

Sixteen out of 483 patients (3.3%) with odontogenic infections showed a septic course during the observation period of 5.5 years. All 16 patients displayed at least one risk factor or co-morbidity, which may have promoted the critical course of their infection with extended hospitalisation and intensive care. In all 16 cases of severe odontogenic abscesses we observed a premolar or molar mandibular focus and identified a need for postoperative respiratory assistance or tracheostomy. This is in accordance with recent literature [10, 17]. We recommend a preoperative CT-scan with contrast agent in cases with septic laboratory signs to obtain an exact overview of the extent of the abscess space. If the expected alleviation after initial surgical treatment with incision, drainage and perioperative antibiotic therapy is delayed, an immediate re-CT examination and targeted re-incision should be initiated without hesitation [6]. In our findings clindamycin demonstrated a reduced susceptibility in all groups of isolated pathogens. Tazobactam and piperacillin showed no resistance to any of the bacteria isolated.

Surgical intervention included incision, drainage, sanitation of the odontogenic focus and antibiotic treatment as first-line therapy [5]. Current literature is controversial on whether irrigative or non-irrigative drainage tubes are superior [2]. From our experience, we prefer high-volume irrigation of the abscess cavity with isotonic saline solution (0.9%) in cases with septic progression. Our findings support this approach. The isolated pathogens of intraoperative swabs showed a typical polymicrobial aerobic and anaerobic spectrum of oral bacteria: *Streptococcus viridans* and *Staphylococcus epidermidis*, *Enterococcus faecalis* were the predominant pathogens [1]. The initial calculated antibiotic therapy should focus on these pathogens to inhibit the risk of infection spreading and lower the risk of possible serious complications. Initiating a pathogen adapted antibiotic treatment as soon as possible with tazobactam and piperacillin in combination as first line medication is preferred. Patients that show CRP values above 200 mg/l and white blood cell counts greater than 19*1000/μl on admission require special attention. Our patients showed a noticeable decrease in their inflammation parameters during the second day after use of tazobactam and piperacillin.

## Conclusion

Odontogenic infections with fulminant progression should be treated according to clinical and imaging data with immediate surgical incision, intensification of intra- and postoperative irrigation, and drainage to eliminate odontogenic foci. If needed, repeat imaging followed by further incisions should be performed. Immediate antibiotic treatment adapted to the antibiogram is of utmost importance.

## Data Availability

All data and materials are accessible on a local server of the Department of Oral and Maxillofacial Surgery of the University Hospital Germany.

## Abbreviations

ARDS: Acute respiratory distress syndrome
CRP: C-reactive protein
CT: Computed tomography
HIV: Human immunodeficiency virus
MODS: Multiorgan dysfunction syndrome
